# Supplementary material for: Light-Sheet Scattering Microscopy to Visualize Long-Term Interactions Between Cells and Extracellular Matrix
Source: Front Immunol. 2022 Jan 28;13:828634. doi: 10.3389/fimmu.2022.828634 (PMC8831865; doi:10.3389/fimmu.2022.828634)
Supplement: Supplementary file 1 [file DataSheet_1.docx]

**Supplementary Information**

**
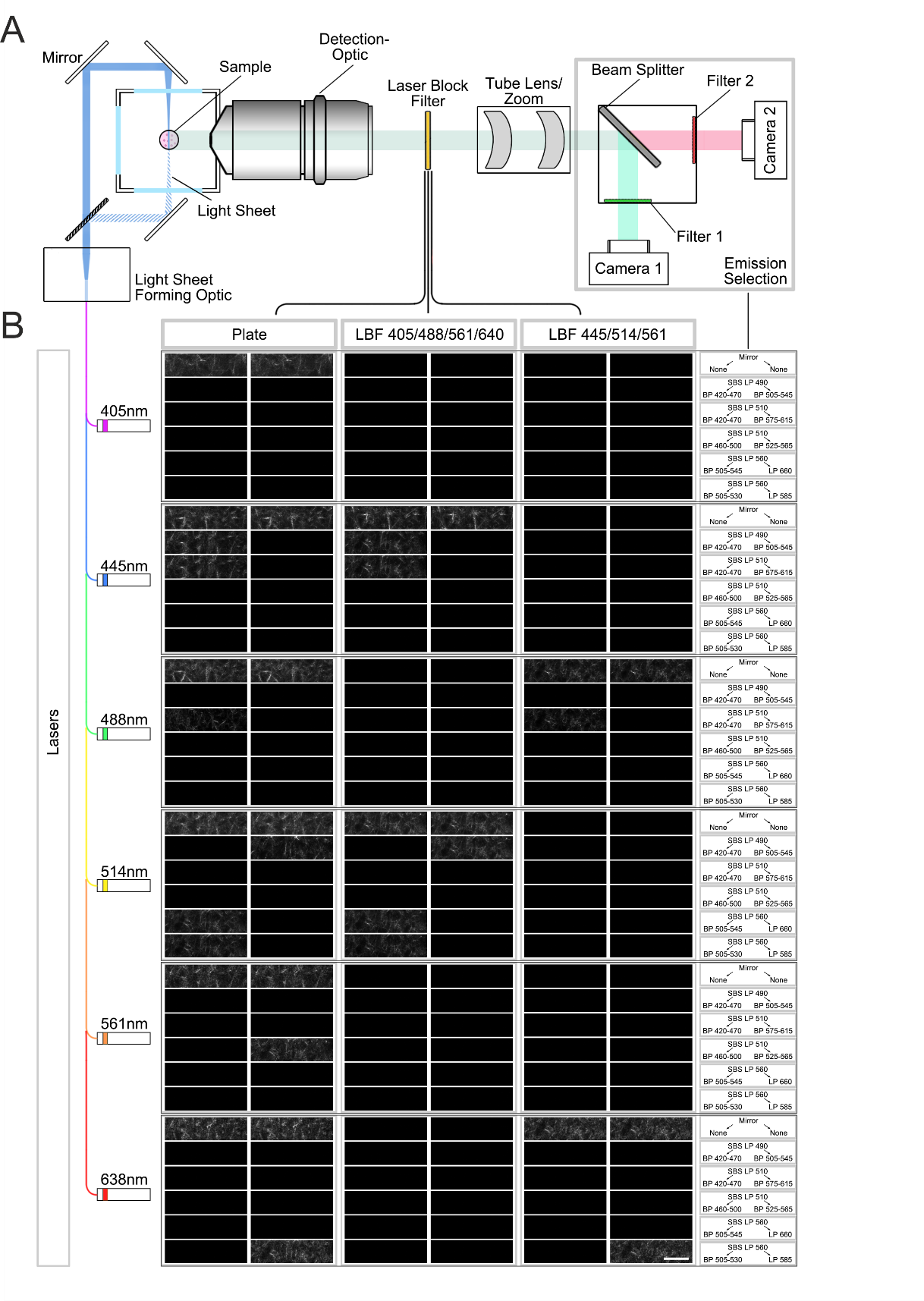
**

**Supplementary Figure. 1 LSSM shows great flexibility regarding wavelength choice.** (A) A brief illustration of the beam path of Zeiss Lightsheet.Z1. Samples are illuminated with a light-sheet. The emission light (fluorescence) and the scattered light is collected by the objective and then filtered by laser block filter. Emission selection modules consist of beam splitters and filters in front of the two cameras to collect different wavelengths of emission light simultaneously. (B) A collagen sample (2mg/ml) was tested in all 216 combination of lasers, laser block filters and emission selection modules. Laser power was set to 0.2%. Exposure time was 149.8 ms. Illumination mode: Single side. Pivot scan: On. Scale bars are 20 μm. Representative images from at least three independent experiments are shown.

**
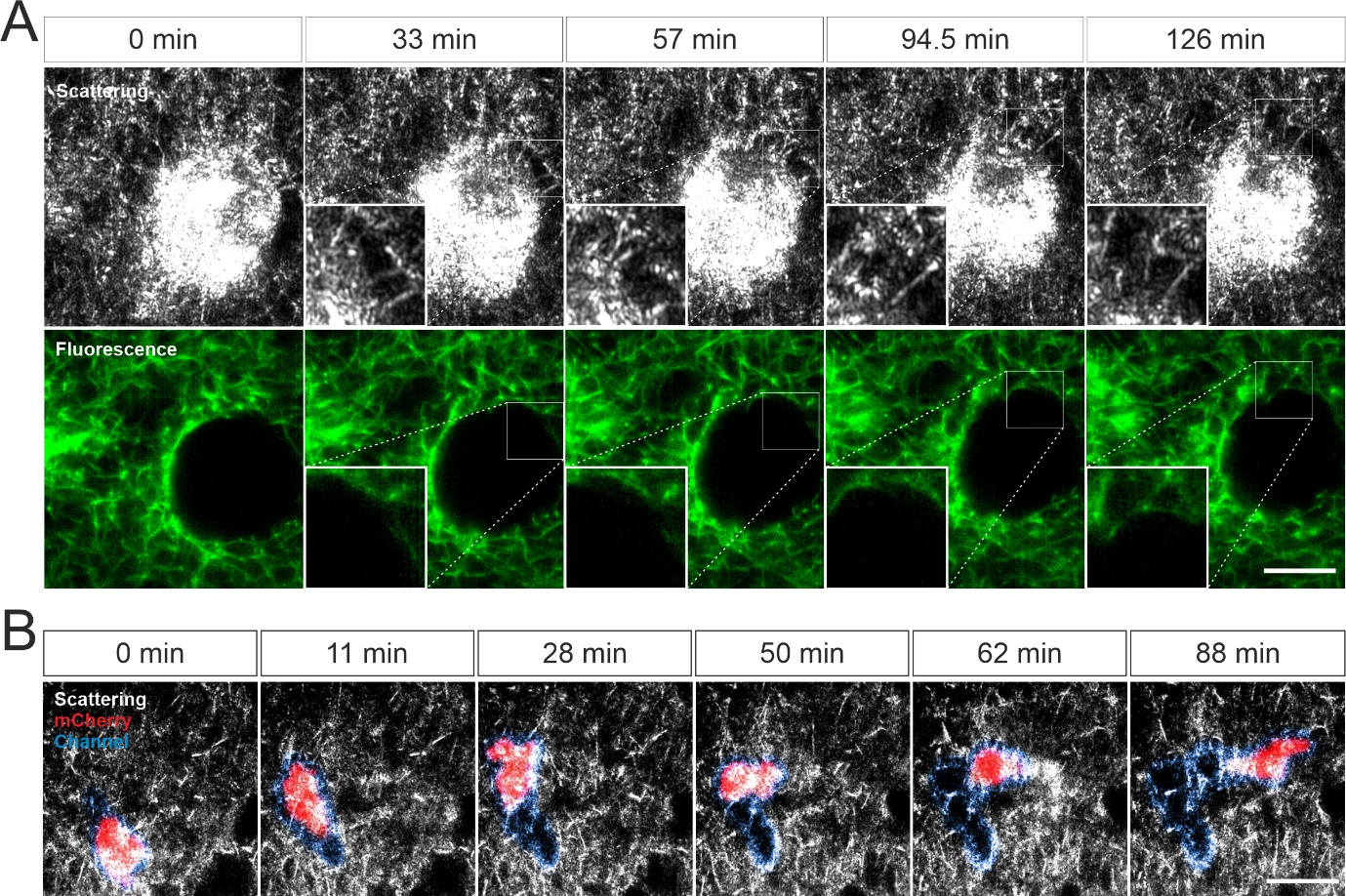
**

**Supplementary Figure. 2 Visualization of ECM-cell interaction using LSSM**. (A) Ultrathin filamentous structures between SK-MEL-5 cells and collagen. Rat tail collagen (2 mg/ml) was fluorescently labeled with Atto 488. Non-fluorescent labeled SK-MEL-5 cells were embedded into the collagen matrix. Z-stacks with a step-size of 0.82 µm for 52 slices were obtained using LSSM and fluorescence modality concurrently at 37ºC every 1.5 min for 2.5 hours. One single slice is shown here. Scale bar=10 μm. (B) Expansion of channels by migrating T cells. Primary human CD8^+^ T cells were transfected with pMax-mCherry and were embedded in the collagen matrices (Bovine collagen, 4 mg/ml). Z-stacks with step-size of 1.5 µm for 70 slices were obtained using LSSM and fluorescence modality concurrently at 37ºC every 1 min for 1.5 hour. Single slice is shown. Scale bar=20 μm.

**Movie legend**

**Movie1-3. Comparison of reconstructed 3D matrix structures obtained by LSSM or fluorescence.** Human collagen (2 mg/ml) was fluorescently labeled with Atto 488. Z-stacks with step-size of 0.421 µm for 101 slices were obtained with LSSM and fluorescence mode concurrently at 37ºC. Reconstructed 3D matrix structures obtained by fluorescence modality is shown in Movie 1, by LSSM in Movie 2. Merged structured from both modalities is shown in Movie 3.

**Movie 4. Ultrathin filamentous structures observed between SK-MEL-5 cells and collagen.** Rat tail collagen (2 mg/ml) was fluorescently labeled with Atto 488. Calcein red-orange loaded SK-MEL-5 cells were embedded in the matrix. Z-stacks with step-size of 0.82 µm for 52 slices were obtained using LSSM or fluorescence at 37ºC every 30 sec for 35.5 min. One single slice is shown here. Time points during the occurrence of ultrathin filamentous structures is displayed slowly.

**Movie 5. Another example for ultrathin filamentous structures observed between SK-MEL-5 cells and collagen.** Rat tail collagen (2 mg/ml) was fluorescently labeled with Atto 488. Non-fluorescent labeled SK-MEL-5 cells were embedded into the collagen matrix. Z-stacks with a step-size of 0.82 µm for 52 slices were obtained using LSSM and fluorescence modality concurrently at 37ºC every 1.5 min for 2.5 hours. One single slice is shown here. Time points during the occurrence of ultrathin filamentous structures is displayed slowly.

**Movie 6. Ultrathin filamentous structures observed with LSSM in 1.4E7 cells.** Rat tail collagen (2 mg/ml) was fluorescently labeled with Atto 488. Non-labeled 1.4E7 human pancreatic beta cells were embedded in the matrix. Z-stacks with step-size of 0.418 µm for 82 slices were obtained using LSSM or fluorescence at 37ºC every 20 sec for 10 min. The upper and lower frame highlights the ultrathin filamentous structures and the displacement of matrix networks by the cells, respectively. One single slice is shown here.

**Movie 7. Visualization of a migrating CD4^+^ T cell in ECM using LSSM.** Primary human CD4^+^ T cells were loaded with CFSE and were embedded in the collagen matrices (Rat tail collagen, 2 mg/ml). Z-stacks with step-size of 1.5 µm for 70 slices were obtained using LSSM (gray) or fluorescence (green) at 37ºC every 1 min for 1 hour. A stack of 3 slices of 3D view is shown here.

**Movie 8. Visualization of a migrating CD8^+^ T cell in ECM using LSSM.** Expansion of channels by migrating T cells. Primary human CD8^+^ T cells were transfected with pMax-mCherry and were embedded in the collagen matrices (Bovine collagen, 4 mg/ml). Z-stacks with step-size of 1.5 µm for 70 slices were obtained using LSSM and fluorescence modality concurrently at 37ºC every 1 min for 1.5 hour. Single slice is shown. Scale bar=20 μm.

**Movie 9. Visualization of cell-induced displacement of collagen fibers.** Non-labeled SK-MEL-5 cells were embedded in rat tail collagen (2 mg/ml). Z-stacks with step-size of 1 µm for 295 slices were obtained using LSSM at 37ºC every 1 min for 2 hours. Pseudo colors are shown. Maximum intensity projection of all slices is shown here.

**Movie 10. Long-term visualization of T cell migration in a collagen matrix.** Calcein red-orange loaded primary human CD4^+^ T cells were embedded in the collagen matrices (Bovine collagen, 2 mg/ml). Z-stacks with step-size of 2 µm for 98 slices were obtained using LSSM and fluorescence modality concurrently at 37ºC every 3 min for 10 hours. Sample drift was corrected. A maximum intensity projection (MIP) of all the 98 slices is shown.

**Movie 11. Long-term visualization of interaction between K562 cells and collagen.** K562 cells stably expressing pCasper was embedded in the collagen matrices (Bovine collagen, 2 mg/ml). Z-stacks with step-size of 2 µm for 75 slices were obtained using LSSM and FRET fluorescence modality simultaneously at 37ºC every 5 min for 20 hours. A maximum intensity projection (MIP) from three exemplary cells is shown. Scale bar=10 µm.
